# Materials Data Science Ontology(MDS-Onto): Unifying Domain Knowledge in Materials and Applied Data Science

**DOI:** 10.1038/s41597-025-04938-5

**Published:** 2025-04-15

**Authors:** Balashanmuga Priyan Rajamohan, Alexander C. Harding Bradley, Van D. Tran, Jonathan E. Gordon, Hayden W. Caldwell, Redad Mehdi, Gabriel Ponon, Quynh D. Tran, Ozan Dernek, Jarod Kaltenbaugh, Benjamin G. Pierce, Raymond Wieser, Weiqi Yue, Kiefer Lin, Jiana Kambo, Christian Lopez, Arafath Nihar, Daniel J. Savage, Donald W. Brown, Hemant Sharma, Brian Giera, Pawan K. Tripathi, Yinghui Wu, Mengjie Li, Kristopher O. Davis, Laura S. Bruckman, Erika I. Barcelos, Roger H. French

**Affiliations:** 1 Department of Computer and Data Sciences, Case Western Reserve University, Cleveland, OH USA; 2 Materials Data Science for Stockpile Stewardship Center of Excellence, Cleveland, OH USA; 3 Department of Materials Science and Engineering, Case Western Reserve University, Cleveland, OH USA; 4 Department of Materials Science and Engineering, University of Central Florida, Orlando, FL USA; 5 FLorida Solar Energy Center, University of Central Florida, Orlando, FL USA; 6 Los Alamos National Laboratory, Los Alamos, NM USA; 7 Argonne National Laboratory, Lemont, IL USA; 8 Lawrence Livermore National Laboratory, Livermore, CA USA; 9 Center for Advancing Sustainable and Distributed Fertilizer Production (CASFER), Lubbock, TX USA; 10 School of Modeling, Simulation and Training, University of Central Florida, Orlando, FL USA; 11 Department of Computer Science, University of Central Florida, Orlando, FL USA

**Keywords:** Research data, Scientific community

## Abstract

Ontologies have gained popularity in the scientific community as a way to standardize terminologies in organizations’ data. Although certain cohorts have created frameworks with rules and guidelines on creating ontologies, there exist significant variations in how Materials Science ontologies are currently developed. We seek to provide guidance in the form of a unified automated framework for developing interoperable and modular ontologies for Materials Data Science that simplifies the ontology terms matching by establishing a semantic bridge up to the Basic Formal Ontology(BFO). This framework provides key recommendations on how ontologies should be positioned within the semantic web, what knowledge representation language is recommended, and where ontologies should be published online to boost their findability and interoperability. Two fundamental components of the MDS-Onto framework are the bilingual package called *FAIRmaterials* for ontology creation and *FAIRLinked*, for FAIR data creation. To showcase the practical capabilities of *FAIRmaterials*, we present two exemplar domain ontologies of MDS-Onto: Synchrotron X-Ray Diffraction and Photovoltaics.

## Introduction

The use of ontologies has gained traction in the scientific community as a universal tool to facilitate data comprehension, analysis, sharing, reuse, semantic data management, and semantic reasoning^1–4^. This is due to their ability as graph-based semantic data models to define and standardize concepts in a given field or domain, group different aliases for the same idea together, and represent complex relationships between these concepts^5^. Ontologies have also become increasingly popular due to their pivotal role in achieving FAIR, an acronym for the foundational data stewardship concepts of Findable, Accessible, Interoperable, and Reusable^1,2^. The FAIR principles are crucial in research, as they ensure that data can be readily discovered, accessed, and utilized by researchers and machines. In the Materials Science domain, where substantial volumes of data are generated daily, sharing data and metadata between cohorts is often challenging due to the absence of standardized vocabularies and unified knowledge within the community^6^. Photovoltaic materials and X-Ray Diffraction research serve as two distinct yet comparable examples where the lack of unified knowledge, in the form of a domain ontology, inhibits effective collaboration within their respective research communities.

In the field of photovoltaics (PV), it is common for PV assets, such as PV Power Plants, to change hands quite frequently, which can cause data and information loss during the transactions. As PV instrumentation is highly non-uniform, it becomes difficult to link raw data to what it actually represents in the physical world. Approaches such as Orange Button^7^ define a taxonomy, and OpenAPI defines a specification^8^ for PV financial transactions, but without significant user support is difficult for a standards-based approach to become widely adopted in the PV research community. Furthermore, photovoltaic modeling depends on a number of different software packages, such as pvlib-python^9^, PVSyst^10^, and SAM^11^, all of which have their own incompatible but required input data formats. Translating between these packages is currently handled on a case-by-case basis, which brings a large maintenance burden upon developers, owners, and researchers. Similarly, photovoltaic researchers perform a wide range of measurements to assess the performance and durability of PV modules^12–16^, using most notably illuminated current-voltage and Suns-*V*_*oc*_ to determine quantities such as maximum power point (*P*_*mp*_), open-circuit voltage (*V*_*oc*_), short-circuit current (*I*_*sc*_), and fill factor (*FF*). However, various photovoltaic researchers use different instrumentation and test conditions (e.g. irradiance, spectral distribution, and temperature) to identify the electrical characteristics of a cell or module. Because these quantities often differ by small amounts, which can be determined by the detailed measurement metadata, it can be difficult to clearly understand which differences area bout the cells, versus which are differences because of the measurement method. Therefore, it is important that the *source* and *conditions* of the measurements are retained. These examples indicate how there is a need for unification of terminology at all levels of the PV supply chain, from research applications to field data collection^17^. Previous approaches to this depended on establishing standards which are challenging to adopt. Here we take an open-source approach^18^, developing and sharing tools to make the task easier. Ideally these packages, frameworks, and tools will eventually become part of the PV community’s solution to these challenges.

Synchrotron X-Ray diffraction (SXRD) is a powerful tool for studying structural properties in different fields such as materials, biology, physics, medicine, and geology. Next-generation synchrotron sources, such as the U.S. Department of Energy’s recently upgraded APS-U^19^, produce highly intense and focused X-Ray beams, which allow researchers to study materials at higher spatial and temporal resolutions by using advanced imaging detectors with small pixel sizes and higher frame rates that generate continuous, large data streams. For example, current data production capabilities at a single beamline in the Advanced Photon Source synchrotron facility at Argonne National Laboratory are 8-10 TB / week (compressed) with a significant increase to 500 TB per week post-upgrade^20^ is anticipated. These data are highly multimodal and include a variety of formats such as images, spectra, diffractograms, and extensive metadata that require flexible processing, access, and long-term data storage solutions. Numerous variable-naming conventions exist that span across the diversity of data formats and sheer volume and variety of data, preventing laboratories from easily understanding the data outputs of each other, making it difficult to achieve automated analysis^21^. Several solutions for data management plans have been proposed in the Photon and Neutron Science committees^22^ (e.g., DMPonline and DMPtool^23^, roaDMaP^24^, and the Data Stewardship Wizard tool^25^) for the long-term maintenance, provenance, and use of the research data in accordance with FAIR principles. However, none of these solutions have achieved the level of adoption necessary to resolve naming convention conflicts between organizations in the SXRD space.

Both of these research communities, PV and SXRD, suffer from the same underlying problem: a lack of terminological consistency in their data and metadata. This problem hinders collaboration, innovation, and data reuse and reanalysis, since it requires research groups time to explain the variables used in their experiments to each other, and, in some cases, data are deemed unusable due to the lack of documentation of variable or terms. Historically, taxonomies were considered the best solution for terminological inconsistency within a scientific domain. However, taxonomies do not have the semantic capacity to fully describe relationships between concepts in domains and therefore restrict the level of reasoning and analysis that teams can gain from adopting the taxonomy^26^.

Ontologies offer an alternative approach to solving terminological consistency by adding a layer of semantic description in the form of non-hierarchical relationships to the concepts they describe^27^. Given their flexible and adaptive structure, ontologies can easily map multiple terms to the same inherent concept and therefore can accommodate varying opinions on terminology. Ontology terms can also be utilized in multiple contexts and across multiple domains without the need to be redefined, promoting terminological consistency and encouraging cross-collaborative research. Furthermore, they can be serialized into popular linked data formats, such as JavaScript Object Notation for Linked Data (JSON-LD)^28,29^, allowing individuals from the scientific community to easily understand and modify the ontology as necessary.

The potential of ontologies to unify knowledge has been proven through the success of the Open Biomedical Ontologies (OBO) Foundry, a collection of ontologies that extensively cover different fields in the life sciences^30^. An example demonstrating this success is the Gene Ontology, which covers the functions of genes and gene products^31^, and became a member of the OBO Foundry in 2004. Since its initial creation, Gene Ontology has become widely adopted in the life sciences community and has been expanded to include more than 45,000 terms and almost 134,000 relationships. It has also been used successfully for many knowledge management and data reasoning tasks, such as designing deep learning models to predict phenotypes^32^, as well as to automatically annotate biological texts^33^. This ontology shows the potential impact that a well-designed and maintained ontology can have on a given field.

Developing an ontology is also the first crucial step toward building knowledge graphs (KG)^34^. A graph is a mathematical concept and data structure that consists of nodes, which represent entities and concepts, and edges, which express the relationships between these entities and concepts^35,36^. Knowledge graphs encode structured and unstructured data in a graph data structure, using ontologies as a schema to organize the information^35^. The entities and relationships between entities are embedded as nodes and edges in the knowledge graph, respectively. The flexibility and extensibility of the graph data structure allow for new data to be incorporated with ease into existing KGs. Knowledge graphs encoded with semantic relationships and data can perform inductive, deductive, and abductive reasoning by deriving implicit knowledge^37^. A knowledge graph is a powerful tool that provides human-contextualized knowledge for intelligent systems and machine learning (ML) or deep learning (DL) models to act and/or advise in domains such as medicine, chemistry, and Materials Science, which is becoming increasingly relevant as the world moves toward incorporating more machines and artificial intelligence (AI) into everyday tasks.

We introduce herein the Materials Data Science Ontology (MDS-Onto) in its version 0.3.0.0 as a low-level ontology and framework developed to address the need for an automated way to build long-lasting and cross-compatible ontologies within the Materials and Data Science domains. MDS-Onto was built by integrating domain-specific ontologies into a unified structure in which terms and concepts between those fields are shared. Each of these ontologies can individually be used for data sharing, data analysis and for model training, validation, and testing, as well as pre- and post-processing of different Materials Science datasets. The ontologies created as part of MDS-Onto can also be utilized by the scientific community on specific domains as a tool to standardize concepts in their field to our Basic Formal Ontology (BFO)-compliant framework. It also enables the creation of JSON-LD templates^28^, following the same variable naming conventions and hierarchical structures that are specified in the ontologies. Those JSON-LD templates can then be used to populate the terms with values, serialize the data, and facilitate the findability and accessibility of the data and metadata.

To create MDS-Onto, we connected specific terms and relationships with preexisting generalized concepts, with the Platform Material Digital core ontology (PMDco)^3,38^ and PROV-O^39^ as a bridge used to connect Material Science terminology up to the ISO Standard BFO^40,41^ and then W3C and Schema.org^42^. We propose a modular and extensive approach to simplify the process of terms mapping and matching to mid- and top-level ontologies. This methodology reduces the learning curve necessary to use the tools and create interoperable ontologies.

It is possible to incorporate and reuse multiple ontologies while building a domain-specific ontology in the case when one ontology does not encompass the needs of the domain-specific ontology envisioned by the domain expert. Other examples of ontologies that can be used to build domain-specific ontologies include the Chemical Entities of Biology Interest (ChEBI) Ontology^43–45^ and the National Cancer Institute thesaurus (NCIt) Ontology^46–48^. ChEBI and NCIt are common ontologies in the chemical and biomedical domains. ChEBI classifies and defines chemical entities such as atoms and molecules that have biological functionality. NCIt provides a wide range of reference terminology and semantic relationships for basic, translational, and clinical research, as well as the bioinformatics infrastructure at the Cancer Institute^47^.

We introduce *FAIRmaterials*, a bilingual R and Python package that allows users to create ontologies with little prior experience. The ontologies created with FAIRmaterials can be used as input to *FAIRLinked*, a Python Package developed to streamline the process of FAIRLinked data creation as JSON-LD files.

In this work, we present the following contributions in the space of ontology development, applied to Materials Science and Applied Data Sciences:

- The MDS-Onto framework for creating ontologies using a simplified and modular approach to promote knowledge unification and long-lasting collaboration within the Materials Data Science community.
- A webpage and tool called MDS-Onto *FindTheDocs*^49^ serving s as a central location for ontologies made through the framework and provides resources for ontology and JSON-LD documentation, visualization and validation.
- A bilingual package (*FAIRmaterials*)^50,51^ to simplify the creation of modular domain ontologies, which overcomes the pitfalls of some of the current state-of-the-art ontology development tools.
- MDS-Onto, a low-level domain ontology composed of multiple Materials and Data Science domain ontologies created via the *FAIRmaterials* package and the MDS-Onto framework, highlighting their practical capabilities and forming the initial basis of the Materials Data Science Ontology.
- *FAIRLinked*^52^, a Python package that works in synchrony with FAIRmaterials to create linked and FAIR data guided by interoperable ontological terms.

This paper is structured as follows. We initially illustrate two application cases in the domains of X-Ray Diffration and Photovoltaics as a means of showing how our package and framework can be used to promote collaboration between labs and streamline data analysis across a range of Materials Science domains. Next, we provide an overview of the current status of Materials Science ontologies and introduce the general framework that we have applied to our ontologies, which extends their usability and usefulness. After that, we analyze current state-of-the-art ontology development tools and introduce a bilingual Python/R package that we have developed to simplify the ontology creation process and a Python package developed to create FAIR data.

MDS-Onto framework and the *FAIRmaterials* and *FAIRLinked* packages show immediate promise toward standardizing the way that Materials Science researchers sort and categorize their data. The development of the MDS-Onto can be used to automate data analysis^53^, perform inductive reasoning^54^, and achieve other analysis tasks that would otherwise be impossible with non-standardized and semantically unstructured data.

## Results: The MDS-Onto Framework Applied to Synchrotron X-Ray Diffraction and Photovoltaics

As the scientific community generates larger quantities of more heterogeneous and multi-modal types of data, there is an increasing need for data to be standardized to enable seamless sharing, analysis, and reuse. The main barrier hindering the interoperability and longevity of experimental data is the variation in how research groups and organizations store, organize, and label their data. Ontologies provide a solution towards unifying the organization and labeling of data by facilitating different aliases for concepts and providing automatic interoperability with other experiment-specific variables. Although the ontology creation process has already been streamlined in specific fields of science, there is still a significant variation in how ontologies are constructed within the field of Materials Science. This variation prevents ontologies from achieving automatic interoperability with one another and therefore hinders effective cross-collaboration between research groups.

With our MDS-Onto Framework, which standardizes key aspects of the Materials and Data Science ontology development process, we showcase the successful implementation of our framework in two case studies: Synchrotoron X-Ray Diffraction and Photovoltaics (PV).

### An Overview of the Synchrotron X-ray Domain

In the field of X-Ray Diffraction (XRD), a synchrotron—or an accelerator—has various path segments where the beam of particles (X-Rays, neutrons, etc.) is guided to an experimental station^55^. These experimental stations are called beamlines. Depending on the size of the facility, there are a varying number of beamlines at a synchrotron source. Even within the same facility, different beamlines have different data collection and metadata recording strategies. These compartmentalized strategies lead to an immiscible pool of data and metadata, making it very difficult for the user to understand what the variables in their data mean. In addition to user-defined variable names, the metadata files are unstructured and often created and recorded in a non-human-friendly format.

Through the MDS-Onto framework, every variable is assigned a human-readable name and mapped to a term from a mid-level ontology. This ensures semantic interoperability with all other variables in the domain. Every variable also has *alt-label* keys in their respective .json files, where the aliases of each variable can be specified. For example, a variable defining the load cell in an experiment could be called load-cll at one beamline and ld-cell at the other. A well-crafted ontology would define this variable in a human-readable format, possibly using the already defined PMDco term “LoadCell,” and then attach the aliases load-cll and “ld-cell as *alt-labels*. The resulting ontology would allow any program written for the variable load-cll to function for alternative names of the variable, and therefore it would allow any code written for data collected at the Advanced Photon Source (APS) of Argonne National Laboratory to also work for data collected at the Cornell High Energy Synchrotron Source (CHESS). Through this example, it becomes apparent that an ontology would make modeling and data analysis easier, as well as promote cross-collaboration between different beamline research facilities.

X-Ray is a subdomain of MDS-Onto that sits below the MDS-charact domain. The collection of subdomain X-ray ontologies (encompassing different techniques that use X-Rays, such as X-Ray diffraction (XRD), X-ray scattering, and X-ray computed tomography (XCT)) form the **MDS-Xray** subdomain ontology.

### Schema of X-Ray Beamline Data

The entire X-Ray subdomain can be divided into the parts specified in the following paragraphs. This encapsulation of data into different subontologies makes the data collection process more efficient. The vocabulary involved in synchrotron-based X-Ray experiments can therefore be represented through the following ontologies, all of which are accessible on the MDS-Onto *FindTheDocs* website.

#### X-Ray Sample Ontology

**Fig. 1:**
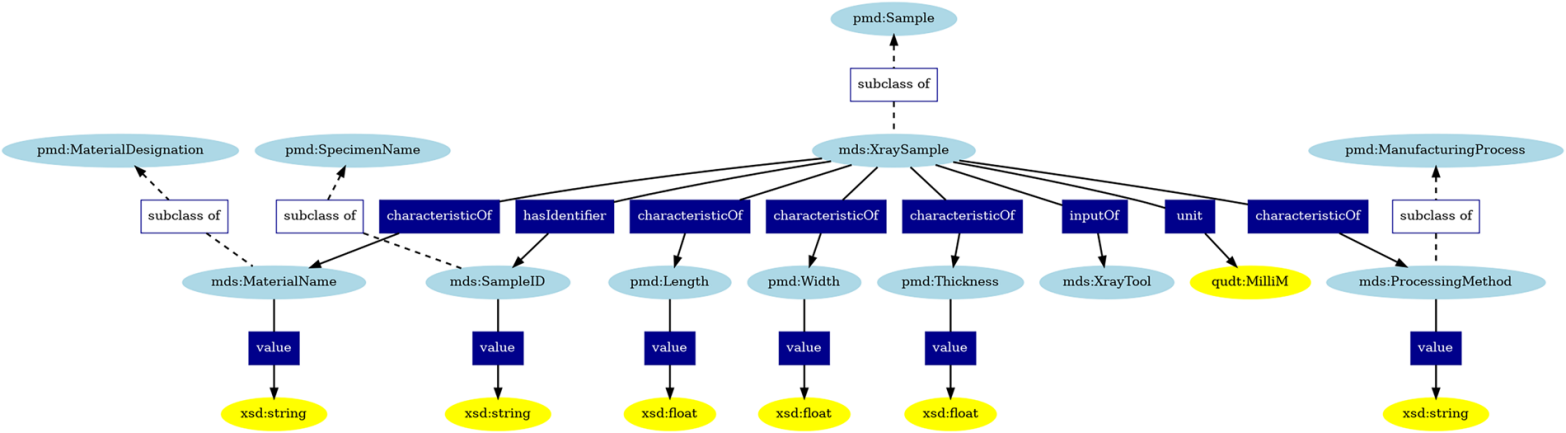
The X-Ray sample ontology. The light blue icons represent ontology terms, with the prefix (i.e., pmd) indicating the ontology that the term was created in. The dark blue rectangles indicate relationships created between entities. The yellow ovals either indicate the type of the value stored in each subclass or the unit that the value is expressed in, with the prefix indicating the ontology the unit definition belongs to or the schema language that the value type is defined in.

A sample is a piece of material that is prepared for testing to collect information about the bulk behavior of the material. We therefore consider the sample as the defined “study object” of the XRD experiments. In the organization of XRD terminology, we encapsulate the information about the sample in its sample ontology. This leads to better “segmentation”, or modularity, of the data and also helps reduce redundancy. Since X-Ray-based characterization techniques are generally non-destructive^56^, several experiments can be performed on a single sample. Therefore, storing the sample information every time is impractical. The X-Ray sample ontology contains all the information about the sample, including the manufacturing process, the material, the phase fractions, the manufacturer, etc. Figure 1 represents the various variables and how they are connected to PMDco.

#### X-Ray Tool Ontology

**Fig. 2:**
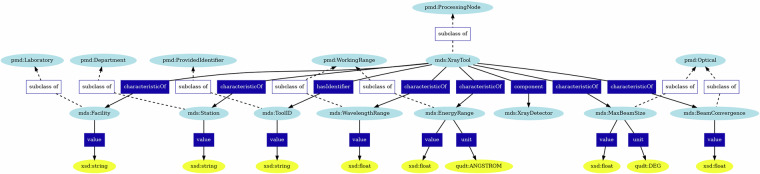
The X-Ray tool ontology. The light blue icons represent ontology terms, with the prefix (i.e., pmd) indicating the ontology that the term was created in. The dark blue rectangles indicate relationships created between entities. The yellow ovals either indicate the type of the value stored in each subclass or the unit that the value is expressed in, with the prefix indicating the ontology the unit definition belongs to or the schema language that the value type is defined in.

To perform a synchrotron-based X-Ray experiment, one needs a beamline or a *tool*, so for example the 1-ID beamline at DOE’s Advanced Photon Source. The tool ontology contains information on where and with what tool the experiment was performed. Figure 2 represents the schema or structure of the X-Ray tool graph. The ontology structure of the X-ray tool helps to FAIRify data collected at different beamlines. For instance, if an experiment is conducted at the 1-ID beamline of APS, the “Facility” key would contain the value “APS”, and the “Station” key would contain the value “1-ID.” Similarly, if the experiment is performed at the FAST beamline at CHESS, the “Facility” key would contain the value “CHESS”, and the “Station” key would contain the value “FAST.”

#### *X-Ray Detector Ontology*

The detector—or camera—collects the diffracted beams and is n essessential part of X-ray-based experiments. A detector ontology stores general information about the detector. This information helps the user determine the information needed about the detector to analyze the collected raw data further.

#### *X-Ray Recipe Ontology*

The recipe is a set of settings and/or steps required to collect a given dataset on a specific tool. It contains information on all the various components used, their values, and configurations used to arrive at the raw data. All of the information a user needs to understand a particular experiment is stored in the recipe ontology. The X-Ray recipe contains information on every stage of the experiment. It is divided into the following subcategories: *Calibration*, *Experiment Type*, *Beamline Configuration*, and *Data-Collection*. The calibration subcategory contains the information needed for calibration of the detector and sample, which is one of the first steps in data analysis. As is evident from the name, the experiment type informs the user of the experiment being performed. The beamline configuration helps researchers consider the information about the beamline tool used for the experiment. This differs from the tool ontology, which contains general information about the tool, including characteristics such as its location and the source’s energy range. In contrast, the beamline configuration contains information about the specific energy or wavelength used for that experiment. Lastly, the data-collection subcategory of the X-ray recipe contains information about the detector parameters used for the experiment, since the detector is the data-collection device.

#### *X-Ray Result Ontology*

Generally, the output of an X-ray experiment is a sequence of images or a movie. The result ontology describes the result of the experiment as well as a log file with the corresponding metadata.

##### *X-Ray Data Pre-Processing Ontology*

All X-Ray experiments have some standard components regardless of the experimental objective. A X-Ray source is needed to bombard the sample with X-Rays. A detector is also needed to capture the reflected or transmitted beams. Some standard processing techniques need to be applied to the primary experimental data that stem from the setup and equipment^57^. These processing techniques might include correcting for the background that is captured by the detector or calibrating the detector with a calibration sample. After a few basic operations, analysis of the experimental data can begin.

These post-processes are extremely important as the final results of data analysis are susceptible to different choices in these corrections. Therefore, unifying knowledge about not only the data, but the entire preprocessing—and analysis—becomes extremely relevant. The post-processing ontology contains information about the corrections applied to the primary data.

#### *X-Ray Data-Processing Ontology*

In the context of an X-ray diffraction experiment, one of the most common (though not essential) post-processing steps is the reduction of 2D raw data into 1D intensity vs 2*θ* plot. This data processing helps quantify the information present in a 2D diffractogram pattern^58^. There are various methods of integrating 2D data into a 1D dataset, and they significantly affect the results of analysis. Hence, the unification of knowledge for the acquisition and analysis of data from an experiment becomes extremely important. The data-reduction ontology provides this unification, keeping track of the steps and methods involved in data reduction.

### An Overview of the Asset Owner’s PV Site & System Domain

Similarly to the field of Synchrotron X-Ray domain, the domain of Photovoltaics also suffers from a lack of unified knowledge because of the variability of PV instrumentation and the different data format requirements of PV-related software. Although there have been previous attempts made towards resolving this issue, such as the Orange Button taxonomy, there is yet to be an approach that has been widely adopted by the PV community. Because of this, the PV Ontologies became the second group of Ontologies that were created through the MDS-Onto Framework. Using the structure of ontologies, we can associate aliases for PV terms to the same concept and establish the semantic relationships between these PV terms. One of the main components of the PV Ontology is the photovoltaic site, which consists of different sub-systems and domains that obey primarily *has-a* relationships^59^. For example, a PV Site *has-a* associated PV System, or a PV module *has-a* PV Cell. It is crucial to retain information at all levels of specificity to gain an accurate understanding of a PV Site’s functioning, which highlights the importance of creating PV Ontologies. Following the same structure of the Synchrotron X-ray domain (and MDS-X-ray), PV is a subdomain ontology of the MDS-BuiltEnv domain ontology. The collection of PV subcomponents (site, system, inverter, battery, charger controller, module, backsheet and cell) for the subdomain ontology PV-Onto.

### Schema of PV Data

Similar to the case of the XRD ontologies, much of the PV domain can be separated into the below parts, the ontologies of which can also be found on the MDS-Onto *FindTheDocs* website domain search page.

#### *PV Site Ontology*

A PV Site forms the highest level of the hierarchy. It represents the physical location where the PV System is installed, and therefore has associated data such as Latitude, Longitude, and Elevation.

#### *PV System Ontology*

A PV Site must have a PV System, and it can have more than one. For example, a Site may have multiple similar, but isolated, Systems that tie in with separate Inverters, or a single, large grid-connected System and an auxiliary research or quality control System.

#### *PV Inverter Ontology*

PV inverters are needed to convert the direct current (DC) power produced by PV Systems into alternating current (AC) power for grid export. A utility-scale System will have many inverters, as each inverter is only rated for a set amount of input DC power before it reaches capacity. When an Inverter reaches its maximum DC power input, the remaining power is curtailed and lost. Knowing the DC power limits of the Inverter is therefore very important for identifying periods of clipping and where additional hardware may be needed.

#### *Storage Battery Ontology*

A PV System might have some type of Storage Battery. Batteries have historically been used in PV systems as a form of emergency redundancy; in this operation mode, the batteries are kept full and only discharged during loss-of-service events. More recently, batteries have become larger, less expensive, and more resilient, making them more suitable for arbitrary shifts in energy availability over time (arbitrage) or as “delayed generators”. Batteries can be filled by PV systems during peak electricity production times, and then deployed at sunrise or sunset to combat the “duck curve” problem of PV generation^60^. Battery systems can be connected directly to PV arrays on the DC side of the system, which improves efficiency and emergency availability at the cost of a complicated and expensive control module, or can be connected after the Inverter on the AC or grid side.

#### *Charge Controller*

As mentioned above, Storage Battery systems require a Charge Controller, which determines when and how batteries are charged or discharged. Charge Controllers can operate either from the electricity grid or can be “islanded”, or isolated from the grid. It is useful for an asset owner to be able to record the details of the Charge Controller, as the field has not yet standardized a system design. Therefore, there is a large variance in the way charge controllers might behave.

#### *PV Module Ontology*

**Fig. 3:**
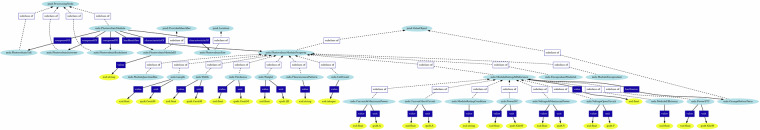
The PV Module Ontology.

The bulk of a PV System’s detailed performance depends on the PV Module used. There are many different brands of PV Modules, and performance depends greatly on many factors, which are recorded in the domain ontology. Module manufacturers tend to have similar, but not standardized, terminology. Module testing agencies such as the California Energy Commission (CEC) tend to develop such terminology as well. Modules undergo qualification and reliability testing, and the parameters of interest are represented within the ontology. The Orange Button taxonomy provides some terminology for PV Modules and PV Inverters, and these terms have been preserved in this ontology and explicitly marked as originating from the Orange Button taxonomy. Figure 3 ilustrates the structure of the PV Module ontology.

#### PV Backsheet Ontology

PV Modules have a Backsheet, which can be a multilayer polymer laminate or a glass sheet, added to the rear side of the PV module. Information about the PV Backsheet is often not included on a Module specification sheet from the manufacturer, so if this information is known, it is valuable to include, as the details of the PV Backsheet can significantly impact how a Module degrades^17^.

#### PV Cell Ontology

A PV Cell is a semiconductor-based device that converts light into electricity and is the smallest unit of a PV Site. Approximately 95% of the photovoltaic cells produced globally are made using crystalline silicon (c-Si) wafers^61^, and these PV Cells are generally identified by the so-called cell technology or architecture, which dictates the manufacturing processes used to produce the cells^62,63^. Older cells are primarily Aluminum Back Surface Field (Al-BSF), while more modern cells are Passivated Emitter and Rear Cell (PERC)^12^. As the technology develops, Tunnel Oxide Passivated Contact (TOPCon) cells and Silicon Heterojunction (SHJ) cells are expected to gain significant market share^61,64^.

**Fig. 4:**
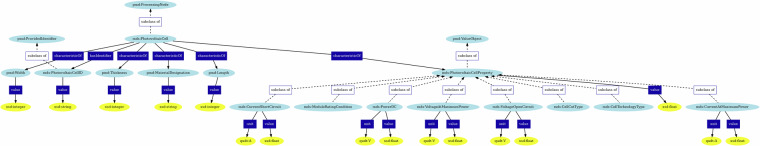
The PV Cell Ontology.

Figure 4 introduces the structure of the PV Cell ontology.

## Discussion

### Impact of Ontologies on X-ray Beamline Data

X-ray ontologies can help unify knowledge about the configuration of an experimental setup to make it appropriate for the material sample being studied. This means that the respective ontology of the experiment would contain information about all the experimental parameters and configurations. For a detector, the ontology would contain all the information about its tilt, center, positioning, etc. Similarly, the sample ontology contains all the information about the sample being studied. Since one could do ten different experiments on a single sample, the user can look at just one file to obtain all the information needed to know about a specific experiment done on a sample. In this case, information about a particular sample is not needed in every experiment. This separation of data and metadata information into distinct ontological groups helps make the data and analysis more manageable and shareable.

The MDS-Onto Framework also helps to manage the analysis of these data. Several studies have used deep learning to help researchers learn from diffraction data^65,66^. It also helps to streamline and standardize statistical processes such as deep learning and neural network training. This, in turn, makes science more reproducible.

### Impact of Ontologies on PV Site & System Data

Ontologies provide a powerful tool for PV power plants, as they enable data to be preserved with the correct identifiers and metadata at all scales, from the Cell to the Site. From an asset owner’s perspective, encapsulating all of this information into an ontology and a few key .json files is valuable, as it provides an easy means for information transfer when an asset changes hands. On the modeling side, asset owners are often concerned with the expected performance of a PV installation; this requires a detailed digital twin of the system to be constructed using FAIR data and metadata that can be accessed through the ontology using the MDS-Onto Framework.

## Methods : A Proposed Ontology Development Framework for Materials Science Ontologies

### The Current Status of Materials Science Ontologies

Ontologies were first recognized in the 1980s as a promising way to make machine-understandable and shareable information within the semantic web^67^. The initial purpose behind creating ontologies was to break down silos through a commonly controlled vocabulary and to provide easy-to-parse semantic information and structure about a certain dataset or domain. Therefore, ontologies were designed to contain information such as the definition of a concept, alternative names for that concept in the literature, a unique Uniform Resource Identifier (URI) to identify the concept and the connection of the concept to other concepts^68^. Due to their malleable and accommodating structure, ontologies have also been increasingly acknowledged for their ability to articulate complex and heterogeneous information, enrich contextual information, and enable the description of intricate and diverse data sources. Due to this flexibility, different pockets within the scientific community began creating ontologies to provide a level of semantic structure and organization to their experimental data^67^.

One of these pockets was the field of Materials Science, where more than 40 ontologies exist today, covering domains ranging from interfaces in batteries to nanoparticles^69^. Although these ontologies successfully define concepts in their respective domains, many of them are isolated ontologies (sometimes referred to as core ontologies) or differ in which ontologies they connect to and, therefore, in how they structure their concepts^69^. This difference in structure prevents these Materials Science ontologies from being automatically interoperable and compatible with one another, hindering simple knowledge unification across the domains that they cover. Therefore, a Materials Science framework with specific ontology creation guidelines is needed to ensure that future Materials Science ontologies automatically reflect this compatibility and interoperability.

Similar frameworks have already been established by different cohorts of the scientific community, and their relative successes demonstrate the impact that a framework can have on the general unification and interoperability of knowledge^30,70^. One of the greatest examples of a successful framework is the OBO Foundry principles, which consists of twenty key principles that cover every facet of ontology development^71^. These principles recommend that (a) relationships used in ontologies be taken from the Relation Ontology (RO), (b) ontologies be specified in a common formal language, and (c) ontologies fully encapsulate their selected domain. Through these principles, over 170 OBO Foundry ontologies have been established for use in biomedical research, and each of these standardizes vocabulary and unifies knowledge in a given biomedical research domain^72^. Since these ontologies were created using the same underlying principles and an agreed-upon structure, OBO Foundry has also been able to create tools to automate ontology workflows and easily compare ontologies for all OBO Foundry ontologies, showing the benefits of creating a framework^73^. Below, we introduce some key recommendations that comprise our MDS-Onto Framework, namely the suggested positioning of all MDS ontologies, what knowledge language representation should be used for MDS domain ontologies, and where they should be published.

## Materials Data Science Ontology (MDS-Onto)

Materials Data Science Ontology, a low-level ontology, sits directly below PMDco and other mid-level ontologies in its specificity level. It unifies concepts from various Materials Science domains by integrating domain-specific terminologies under the same overarching ontology. Even though PMDco is primarily used to map the core terms of MDS-Onto, other mid-level ontologies that are BFO-conformant can be used to map terms and concepts if terms are not available in PMDco. By matching our terms to BFO, we ensure that our ontology is positioned under schema.org and the semantic web, allowing developers to check term and relationship mappings with other registered ontologies and find resolutions early (usually by adopting the other term). This process is referred to as ontological alignment and expands out of the single domain to all concepts, and even into other domains.

### Purpose of Modular and Extensive Approach

**Fig. 5:**
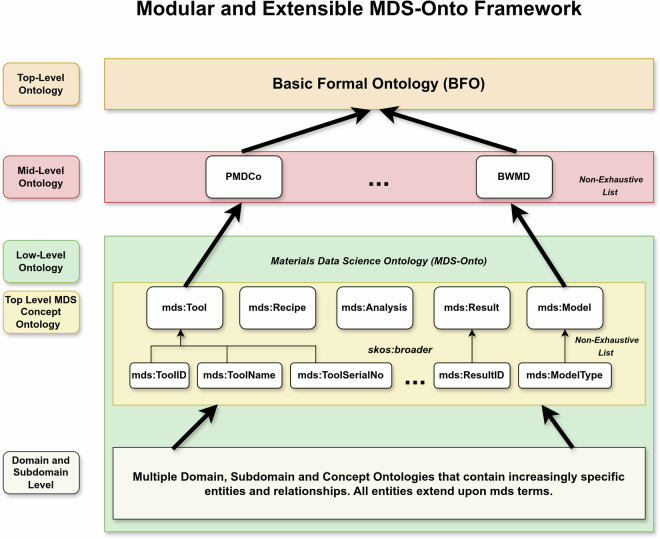
Modular and Extensive MDS-Onto Framework.

Using PMDco (Platform Material Digital Core Ontology) as the main semantic bridge between the MDS (Materials Data Science) Ontology (MDS-Onto) and upper-level ontologies such as Descriptive Ontology for Linguistic and Cognitive Engineering (DOLCE)^74^ or BFO may introduce significant challenges. Different scientific domains present divergent concepts and relationships that may not naturally extend from those defined in PMDco. To overcome the direct dependency of PMDco terms to align to MDS concepts, we propose a modular approach and extensive framework designed to achieve interoperability through ontological mappings. Figure 5 illustrates the general structure of the MDS-Onto Framework with its hierarchical components. At its core, the framework utilizes BFO as the upper-level ontology to which MDS-Onto is aligned.

The middle layer, known as the bridge layer, hosts one or more mid-level ontologies such as PMDco, QUDT (Quantities, Units, Dimensions, and Types Ontology)^75^ that serve as semantic intermediaries between user-defined terms and relationships within MDS and the abstract concepts in BFO. This layer ensures that specialized concepts in MDS-Onto are seamlessly aligned with the overarching framework of BFO, thus enhancing semantic consistency. Consequently, the framework facilitates the effective interoperability of diverse datasets while promoting coherence and extensibility between scientific disciplines. The MDS Ontology (MDS-Onto) constitutes a low-level ontology structured into multiple layers, with each higher layer representing increasingly abstract generalizations compared to the layers below.

The top-level MDS Concept Ontology extends the terms and relationships provided by the ontologies within the bridge layer. This layered approach enables modularity and extensibility by isolating changes within specific layers, eliminating the need for widespread modifications across the entire stack when updates are made. All subdomain and domain ontologies in MDS-Onto reuse or extend mds terms and relationships exclusively to maintain consistency across the stack. Certain external ontologies, such as QUDT for specifying units and SKOS^76^ for alternative labels, are allowed for well-defined purposes. By restricting the use of external ontologies to targeted tasks, such as units or labeling, MDS-Onto maintains its modular integrity while benefiting from established, widely adopted standards. Terms such as mds:Tool and mds:Recipe not only extend concepts from bridge layer ontologies but also serve as essential categories for grouping entities defined within the Top-Level MDS-Concept Ontology.

**Fig. 6:**
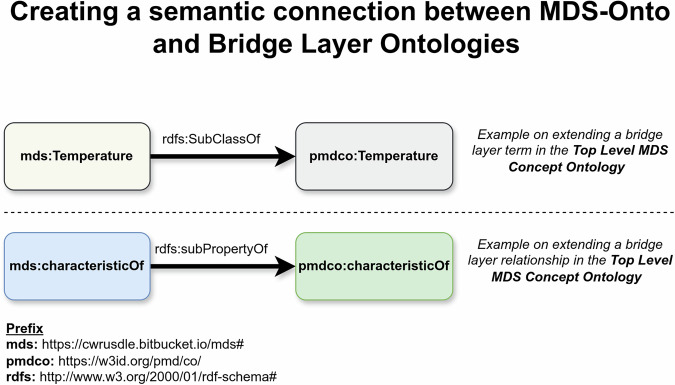
Modular Approach: Connecting MDS-Onto to PMDco.

Figure 6 illustrates the process of establishing a semantic connection between MDS-Onto and a mid-level ontology. While this approach may initially seem to introduce redundancy by replicating terms, it ensures that all domain and sub-domain ontologies are built upon MDS terms and relationships, avoiding direct dependencies on external ontologies. As a result, updates-such as changes to PMDco’s entities or relationships can be managed by modifying only this semantic connection within the Top-Level MDS-Concept Ontology. This localized adjustment prevents widespread alterations across the system, enhancing the framework’s modularity, flexibility, and maintainability.

Domain experts often create new ontologies and integrate them into MDS-Onto without adequately considering the reuse of existing terms already defined in other ontologies. To address this challenge, MDS-Concept ontology proposes to identify and promote the reuse of common concepts within MDS-Onto. By reducing duplication and enforcing term reuse, this approach enhances consistency and efficiency across ontologies. When domain ontologies and subdomains are finalized, they are merged and added to a new version of MDS-Onto. In the MDS-Onto Framework, sub domain level terms are labeled following  community stardards guidelines such as the recommendations from Research Data Alliance Persistent Identification of Instruments Working Group RDA (PIDINST) on how to publish persistent identifiers for instruments^77^. 

**Fig. 7:**
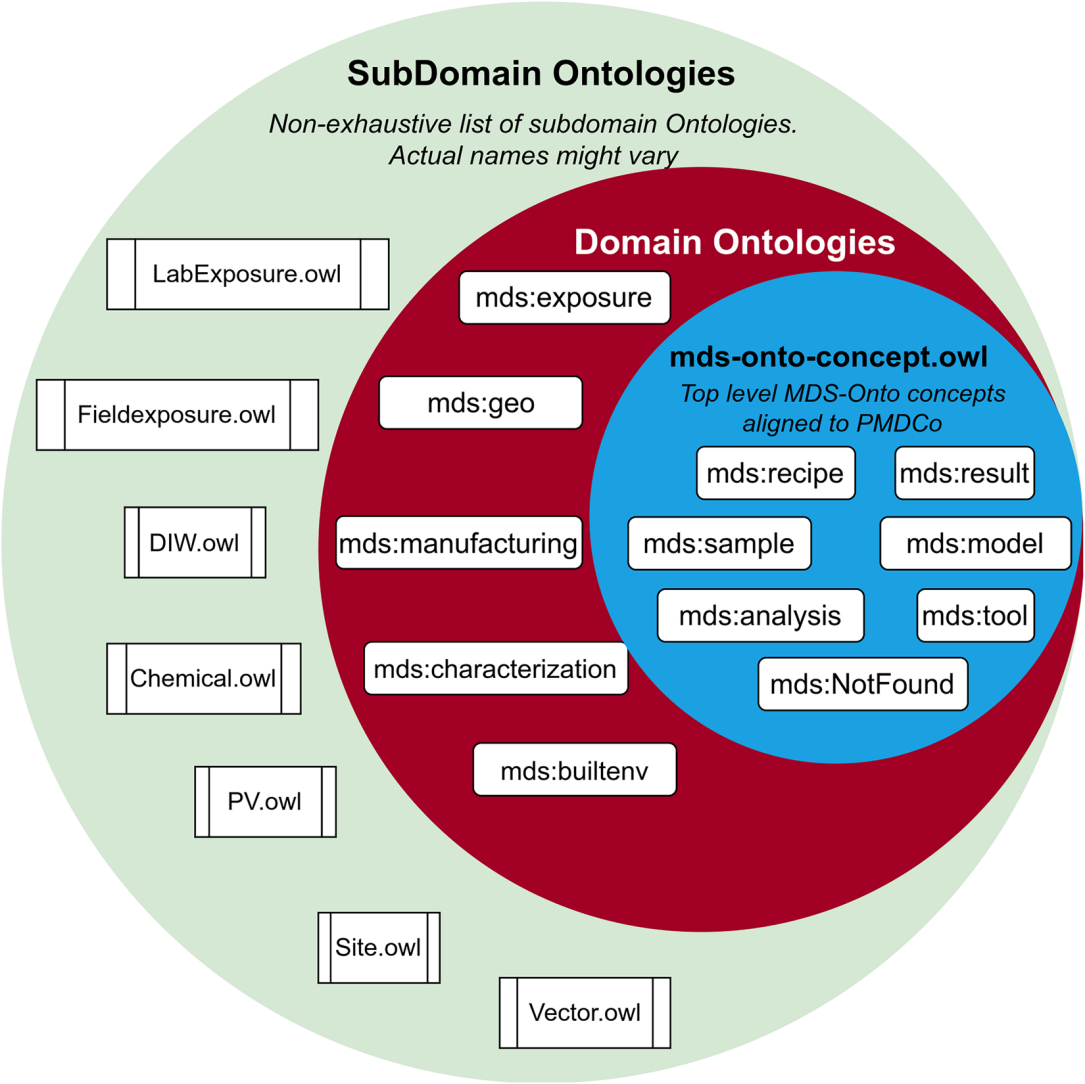
Reducing conceptual duplicates using concept ontologies.

Figure 7 shows that the current MDS Ontology is organized into several domains and subdomains. Each subdomain ontology is nested within a specific domain, such as mds:manufacturing, mds:exposure, mds:characterization, mds:geo, and mds:builtenv.

### Constructing Domain Ontologies through the MDS-Onto Framework

#### Resource Description Framework

Apart from its position within the semantic web, one of the most important aspects to consider when creating MDS subontologies is that they are expressed in a formal knowledge representation language that optimizes machine readability, data interoperability, and data integration. Given these priorities, ontologies are often described using a knowledge representation language called the Resource Description Framework (RDF)^78^. The RDF describes knowledge using textual triples in the following sequence: a subject, a predicate, and an object. In this textual structure, the predicate explicates the relationship between two entities, that is, between the subject and the object. The RDF became a W3C recommendation for data interchange in 2004^79^, with an update to v1.1 in 2014^80^, and has since become an essential component of ontology development due to its natural facilitation of ontology merging and its inherent compatibility with ever-evolving data schemas. The Resource Description Framework Schema (RDFS)^81^ and its recent extension to RDF-Star^82^ extend the RDF with a standardized vocabulary for common relationships between resources, including terms to define hierarchical relationships and terms to denote the domain and range of relationships. Although RDFS and RDF-star greatly expands the relational and terminological complexity that can be incorporated into an ontology, they still lack the terminology to accommodate highly complex relationships between concepts and properties. The **Ontology Web Language** (OWL)^83^ was therefore created in 2004 and updated to OWL2 in 2012^84^ to further expands the progress of RDFS by facilitating symmetrical and transitive relationships, disjointedness among classes, and other crucial logical axioms. Using RDF in conjunction with RDFS/RDF-star and OWL2, Materials Data Science researchers have the necessary vocabulary to fully describe complex domains. For these reasons, we chose to incorporate all of these into our MDS-Onto framework.

#### MDS-Onto *FindTheDocs*

**Fig. 8:**
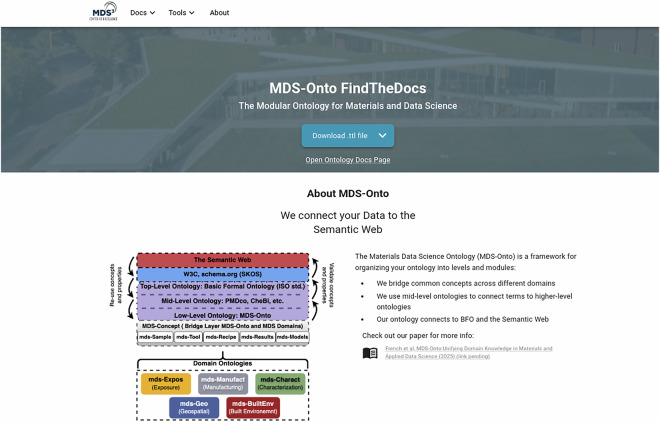
Home page of the MDS-Onto *FindTheDocs* website. The website consists of all of the MDS domain ontology files in different syntaxes along with corresponding documentation, links to resources to aid ontology visualization and analysis, and documentation on how to use the *FAIRmaterials* package.

Once an ontology has been created in the proper semantic web positioning and with the aforementioned knowledge representation language specifications, it is pivotal that the ontology be published so that other researchers can suggest modifications and incorporate it into the semantic management of their own experimental data. The *MDS-Onto FindTheDocs* website, as illustrated in Fig. 8, performs this task by providing an open and easy-to-navigate interface that allows users to search for any ontology that has been created through the MDS-Onto Framework. In addition to providing every MDS ontology in multiple syntaxes, the website also includes documentation for terms in every ontology, as well as a static visualization of the ontology. Furthermore, the website consists of a variety of resources for ontological analysis and visualization, including tools like **WebVOWL**^85^ and **JSON-LD Playground**^86^, and extensive documentation on how to use the *FAIRmaterials* package, which will be discussed further in Section 3. This website has been made available^87^ at https://cwrusdle.bitbucket.io/.

#### MatPortal

We have published the latest version of the entire MDS ontology to the MatPortal ontology repository^88^, and the Industry Portal^89^. MatPortal and Industry Portal are publicly accessible repositories with a collection of over thirty Materials Science Ontologies and an arsenal of tools for ontology discovery, analysis, and comparison. These Ontology Portal websites are based off of the open-source BioPortal^90^ website and its network of connected ontology portals, which consists of more than 70 ontologies and engages the biomedical community to review and help improve ontologies through its ontology development features. By publishing our ontology to MatPortal and IndustryPortal, we are allowing the Materials Science community to review our ontology and identify and assess potential mappings between our terms and terms from other ontologies. This ensures that we remain in sync with the rest of the Materials Science community when it comes to ontology maintenance and evolution.

#### The Relationship between MDS-Onto and FAIRification

FAIRification is a method of standardizing the collection and storage of experimental data to ensure that the said data are findable, accessible, interoperable, and reusable^1,2^. Continual efforts are underway to FAIRify different forms of data to allow for smooth cross-collaboration between scientific communities. These efforts include making all data readily Findable by implementing unique identifiers across data portals, ensuring efficient Access through standardized interfaces for seamless sharing, establishing Interoperable links to connect disparate datasets and enhance their usability across various software tools, and promoting data Reuse by providing comprehensive licensing information. Detailed descriptions and industry-approved definitions of the FAIR data standard are listed on the Open Source FAIR Cookbook website^91^, providing more clarification on what it means for data to be FAIR^92^. While ontologies do not directly FAIRify data themselves, they are an integral step towards data FAIRification. Ontologies are most directly associated with the Interoperability aspect of FAIRification, as they detail data and metadata in a standard knowledge representation language and provide a framework for automatically connecting two data sets whose variables are mapped onto the ontology. Ontologies also create unique resource identifiers for all concepts and instantiations of concepts, and therefore satisfy a key component of Findability. However, these unique resource identifiers are not automatically findable by machines since the ontology data and corresponding identifiers are not necessarily entered into a searchable resource. Therefore, more effort is needed to fully satisfy the Findability aspect of FAIR. Additionally, since ontology files are not automatically made available to others via a standard communication protocol, there are additional steps needed to make experimental data accessible through an ontology. Lastly, while certain relationships between ontologies can easily allow researchers to attach detailed provenance to data and their corresponding metadata, data usage licenses are still necessary to satisfy the reusability component of FAIR^93–97^. Hence, ontologies should primarily be viewed as a useful tool towards achieving FAIR data rather than as an automatic indicator of FAIRified data.

### Simplifying the MDS-Onto Creation Process with the *FAIRmaterials* R & Python Package

This section outlines the methodology utilized in the development of *FAIRmaterials*^98^. *FAIRmaterials* is a bilingual package in R and Python that translates several .csv files from a public .csv template, which can be found here, with ontology terms and corresponding data in RDF triples. The three main outputs that result from running the package include Turtle (/texttt.ttl) and JSON-LD files storing the translated RDF triples, as well as a graph visualization of the RDF triples (saved as an .svg in R and a .png in Python).

The Python version also includes a fourth output. It generates documentation for the output ontology as a .html file. The *FAIRmaterials* package is designed to be a simple and user-friendly tool to create and visualize ontologies, allowing researchers of all levels of ontology experience to engage in knowledge management. This tool makes it easier for individual researchers to create ontologies by offering an alternative to complex traditional ontology editing platforms such as **Protege**^99^. *FAIRmaterials* simplifies ontology development through a .csv-based template system, which has well-documented instructions and is intuitive to use. The package’s conversion of simple and structured .csv inputs into rich, well-defined ontologies facilitates the creation of ontologies to improve FAIRification^1^ of research data.

The RDFLib library of W3C supports the capabilities of the package’s R and Python versions by programmatically creating and populating ontologies^100^. **RDFLib**^101,102^ also allows serialization into many syntaxes, including Turtle and JSON-LD, for diverse user needs. The package not only helps generate ontologies, but also provides tools for static visualizations, and the generation of detailed .html documentation (in Python) improves both the readability and usability of ontologies.

*FAIRmaterials* is designed to simplify ontology development and address the lack of standardized vocabularies in many domains that hinder data sharing and interoperability. Through a user-friendly .csv-based template system, *FAIRmaterials* enables researchers of all levels of ontology experience to design and expand ontologies efficiently. This approach in combination with additional features of the package, such as automated ontology merging, static visualizations, and comprehensive documentation, increases its utility in implementing the FAIR principles. Major benefits of FAIRlinked is the ease of creating baseline domain ontologies. We believe that using a csv-based template with a modular approach for terms mappings to mid level ontologies lower the barrier for usage as domain experts can choose to map their terms directly to MDS-Concept, minimizing the efforts required for terms alignement. 

### FAIRLinked: Creating Ontology Aligned FAIR Datasets

FAIRLinked^52^ is a Python package that transforms research data into FAIR-compliant Linked Data. By utilizing the RDF Data Cube Vocabulary, using a measure dimension approach, and mapping data to low-level ontologies (e.g., MDS-Onto), FAIRLinked fosters data interoperability and reusability^103^. This focus on semantic data management and enhancement and FAIR compliance sets FAIRLinked apart from other data management tools, making it especially appropriate for research scenarios that require standardized ontology-aware data publishing. Designed to work alongside researchers in a variety of data generation contexts, ranging from laboratory experiments to observational workflows, such as photovoltaics or geospatial studies, FAIRLinked prompts users with questions before and after running an experiment to facilitate converting tabular experimental data and metadata into an ontology-backed RDF dataset. Users can fully utilize this package by supplying ontologies that adopt frameworks similar to MDS-Onto for scientific disciplines beyond Materials Data Science. In the context of Materials Data Science, MDS-Onto aims to represent its diverse domains and subdomains comprehensively. The primary goal of FAIRLinked is to bridge the gap between ontologies and datasets, enabling data to be shared, semantically reasoned over, and integrated into automation pipelines. These pipelines transform raw data into semantically enriched, FAIR-compliant and AI-ready datasets.

**Fig. 9:**
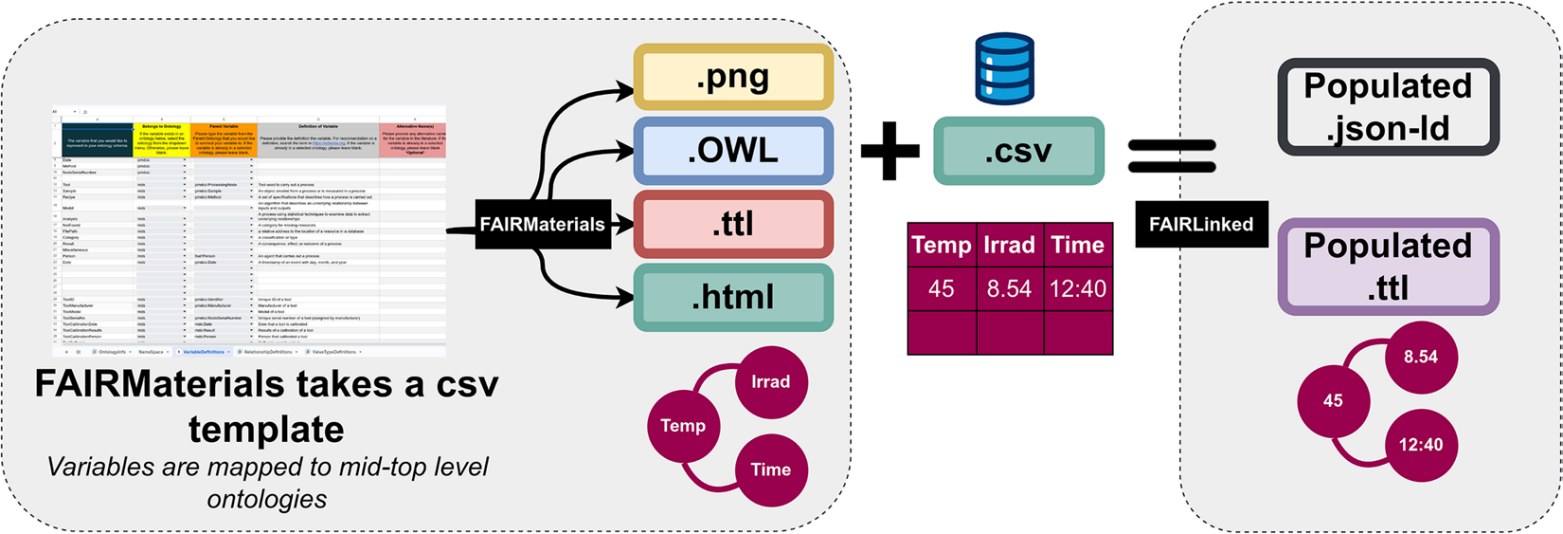
FAIRmaterials and FAIRLinked workflow. In the input layer, the user fills in the .csv template, mapping terms, and relationships to mid-level ontologies, and runs the *FAIRmaterials* package to generate three outputs: JSON-LD empty templates, ontologies (.owl), and a .png for ontology visualization. In the subsequente step, domain Ontologies created with are used to create dataframe templates with interoperable ontological variables. When the dataframe is populated with data is feed into FAIRlinked that outputs populated JSON-LD and .ttl files.

Figure 9 illustrates the general framework of FAIRlinked and FAIRmaterials, which work side by side to create FAIR data. FAIRlinked takes as input MDS-Onto and domain ontologies files to create a dataframe(.csv templates) with variables(column names) extracted from the domain ontologies files that have been modularized to MDS-Onto Ontology. The template is populated with data and is fed into FAIRlinked to generate JSON-LD and turtle files populated with data. Users can specify a single JSON-LD file to be created per dataset or a JSON-LD file per row, depending on the application and user case. By creating JSON-LD files that have unique and parseable fine names, one can easily write scripts to find, access, read, and integrate it to data workflows. It promotes significant advances by creating more reliable, reproducible and efficient research, where data and metadata are monitored throughout their lifecycle and easily integrated into data science pipelines and workflows.

## Community Engagement with MDS-Onto

MDS-Onto Framework is an open-source project with an ensemble of user-friendly and complementary tools available to the scientific community to use in their respective research fields and allow them to contribute to its core development in domain-specific applications. MDS-Onto have a governance structure based on core developers and domain experts leads that oversee and monitor the developement of domain ontologies and creation of FAIR data of research teams in their respective areas. Domain leads responsibility is to ensure quality requirements and consistency of terms and ontologies created within the MDS-Onto Framework as well as enhancing the ongoing collaborations and forming new partnerships.

Domain leads closely interact with the MDS-Onto Framework leadership and core development teams to ensure the transfer of knowledge and that new progress are being properly shared. The MDS-Onto core team often promotes workshops and we host biannual meetings with collaborators to illustrate new developments and foster new partnerships. We also have ongoing projects where domain leads or a core member of development teams directly participate in the research to facilitate the ontology and fair data creation and ensure that data are being properly managed in all its lifecycle.

## Data Availability

All ontology files (.owl and .ttl), supportive documentation (.html) and .json files can be accessed and downloaded from https://cwrusdle.bitbucket.io/) in the tab “Domain Files” in its current version (0.3.0.0). The ontology files are also available on MatPortal (https://matportal.org/ontologies/MDS) and Industry Portal (https://industryportal.enit.fr/ontologies/MDS) and have been published on osf^104^. The current modular MDS ontologies on version 0.3.0.0 are: PV Battery, Cell, Charge Controller, Module, Site, Interdigitated Combs, Surface Insulation Resistance (SIR), IV Curve, Atomic Force Microscopy, Fractography, Profilometry, Pyrometry, X-Ray diffraction and FTIR. Files can be accessed and downloaded directly from the MDS-Onto *FindtheDocs* webpage. New ontologies have been verified and validated for different domain experts in the particular field.

## Code availability

The *FAIRmaterials* Python package can be easily accessed from the Python Package Index (PyPI)^105^. To install the package, simply search for “FAIRmaterials” on the PyPI website^50^ and follow the instructions provided for installation. The package can also be installed with the following Python command:

pip install FAIRmaterials

The *FAIRmaterials* R package can be easily accessed on the Comprehensive R Archive Network (CRAN)^106^. To install the package, simply search for “FAIRmaterials” on the CRAN website^51^ and follow the instructions provided for installation. The package can also be installed with the following R commands:

install.packages("FAIRmaterials") library(FAIRmaterials)

The package includes comprehensive documentation, including detailed usage examples. These can be found in the packages vignette available on the “FAIRmaterials” page on CRAN found^51^. You can also access the package vignette after installation using the following R command:

vignette("FAIRmaterials")

More documentation can be found after the installation using the R command:

?process_ontology_files

The code for both versions can also be accessed through a public GitHub^104^, with more documentation for the packages. The *FAIRLinked* Python package can be access and installed on PyPI^52^ It can also be installed with the command:

pip install FAIRLinked
